# Necrotizing periodontitis or medication-related osteonecrosis of the jaw (MRONJ) in a patient receiving Bemcentinib—a case report

**DOI:** 10.1007/s10006-020-00851-w

**Published:** 2020-05-21

**Authors:** Caspar V. Bumm, Matthias Folwaczny, Uta C. Wölfle

**Affiliations:** Department of Conservative Dentistry and Periodontology, University Hospital, LMU, Goethestraße 70, 80336 Munich, Germany

**Keywords:** Bemcentinib, Necrotizing periodontitis, Medication-related osteonecrosis of the jaw, MRONJ, AXL inhibitor, Acute myeloblastic leukemia

## Abstract

Bemcentinib is a newly developed AXL inhibitor that is currently under investigation in phase II trails for the treatment of acute myeloblastic leukemia (AML). Clinical and radiographic findings in this case were very similar to cases of MRONJ in patients receiving Sunitinib or other anti-angiogenetic substances, assuming that Bemcentinib may cause similar oral side effects. We present a male 81-year-old patient with a manifestation of alveolar bone necrosis at the central upper incisors following a 2-month regimen with the AXL-inhibitor Bemcentinib, administered for the treatment of secondary acute myeloblastic leukemia (sAML). Due to the duration of less than 8 weeks, the osteonecrosis was diagnosed as necrotizing periodontitis, but the intraoral clinical and radiographic findings were also compatible with the differential diagnosis of medication-related osteonecrosis of the jaw (MRONJ, stage II). Following to discontinuation of Bemcentinib, the affected bone was surgically revised including the removal of a demarcated bone sequester under preventive antibiotic treatment (metronidazole 400 mg t.i.d.). We hypothesize that Bemcentinib might increase the susceptibility for osteonecrosis of the jaw, probably related to its antiangiogenic effects and the resulting modulation of host immune response. Based on the current observations, it can be assumed that oro-dental health might be significant also prior and during treatment with Bemcentinib for the prevention of MRONJ.

## Background

The idea of developing an extensive arsenal of active substances for targeted and individual cancer therapy has produced an array of anti-angiogenetic active substances, e.g., Sunitinib [1–4], that might induce medication-related osteonecrosis of the jaw as a side effect. Bemcentinib (BGB 324), currently under investigation in phase II trails (ClinicalTrials.gov/Identifier: NCT03965494, NCT03824080, NCT03654833, NCT03649321, NCT03184571, NCT03184558), is targeting AXL with additional antiangiogenic effects [5]. Herein, we report a case of medication-related osteonecrosis of the upper jaw after Bemcentinib regimen for the treatment of secondary acute myeloblastic leukemia. Clinical and radiographic findings were very similar to cases of MRONJ as previously described using Sunitinib [1–3] or other anti-angiogenetic substances [6, 7]. We show clinical findings and review the literature.

## Case presentation

An 81-year-old male patient with a diagnosis of secondary acute myeloblastic leukemia (sAML) treated with the AXL inhibitor Bemcentinib for 2 months as part of a phase II trail was referred to the outpatient clinic of the Department of Conservative Dentistry and Periodontology, University Hospital Munich due to exposed alveolar bone of the upper anterior jaw persisting for approximately 4–5 weeks in June 2019. Despite discontinuation of AXL inhibitor therapy immediately upon first manifestation, the bone exposure remained unaffected. Besides general fatigue, the patient reported occasional pain in the area of exposure during mastication, whereas other signs of inflammation, such as fever or lymphadenopathy, were not observed. Secondary findings were atrial fibrillation, hypertension, aortic aneurysm, and cholelithiasis. Antihypertensive therapy was conducted with bisoprolol (1.25 mg o.d.), torasemid (10 mg o.d.), and candesartan (8 mg o.d.). In addition, the patient received apixaban (2.5 mg b.i.d.) for the prevention of ischemic stroke. According to anamnesis, the patient was currently not undergoing dental treatment and no oral examination had been performed prior to initiation of treatment with Bemcentinib. Preliminary clinical or radiological dental findings were not available. The patient did never receive radiation therapy of the head and neck region.

**Fig. 1:**
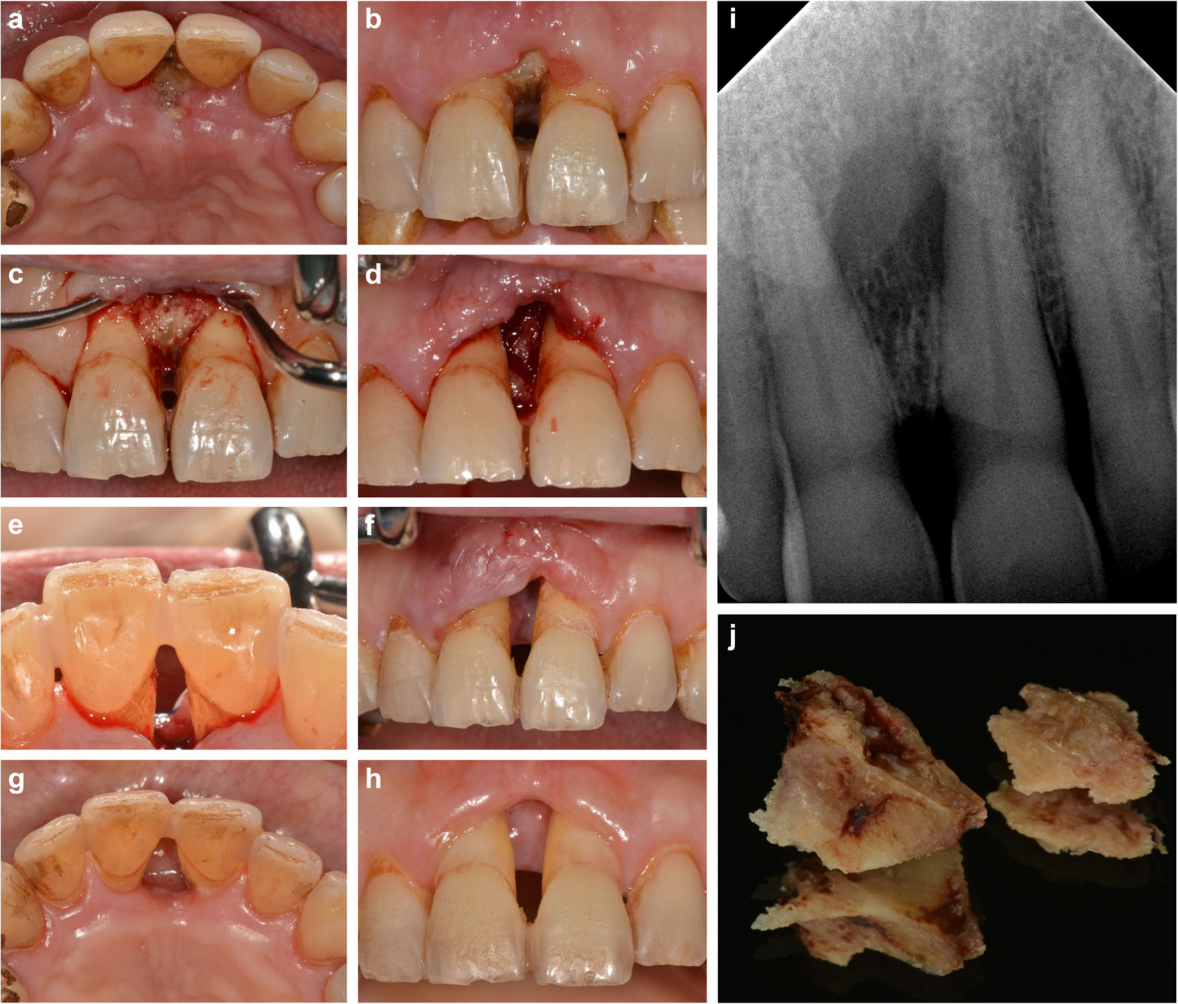
**a**, **b** Intraoral situation with exposed bone between incisors 11 and 21. **c** Surgically depicted sequestrum. **d** Situation after sequestrotomy. **e**, **f** Intraoral situation 7-day follow-up. **g**, **h** Intraoral situation 36-day follow-up. **i** X-ray of incisors 11, 21. **j** Surgically removed sequestrum, divided oro-vestibularly

Clinical examination, including a standardized dental examination, as well as a localized periodontal examination, including the central maxillary incisors, was carried out. In order to avoid excessive bleeding resulting from AML-associated thrombopenia and drug-related anticoagulation, a periodontal examination of the remaining dentition was not conducted. However, a generalized accumulation of hard and soft dental plaque reflected the patient’s inadequate oral hygiene; thus, a pre-existing periodontal disease could not be excluded. Focusing on the maxillary anterior dentition, the interdental gingival tissue of the central upper incisors presented with significant ulceration causing extensive exposure of the crestal parts of the interdental bone. The margins of the soft tissue defect showed minor swelling and redness. The exposed bone appeared necrotic due to grey discoloration and was mobile upon gentle probing indicating partial demarcation and sequestration. Both incisors showed gingival recession defects > 6 mm at the mesial aspects of the tooth root (Fig. 1a, b). The probing pocket depth was 12 mm at the mesio-buccal and the mesio-palatal sites. Apical radiograph of the relevant area revealed a c-shaped radiolucent area of 5 mm in the middle third of the interdental osseous tissue between the central incisors compatible with a zone of demarcation (Fig. 1i). Based on the clinical and radiographic signs and the overall duration of only 4–5 weeks, a diagnosis of necrotizing periodontitis (NP) with the differential diagnosis of MRONJ (stage II) according to the AAOMS [4] and the indication for the surgical removal of the osseous sequester was made.

**Fig. 2:**
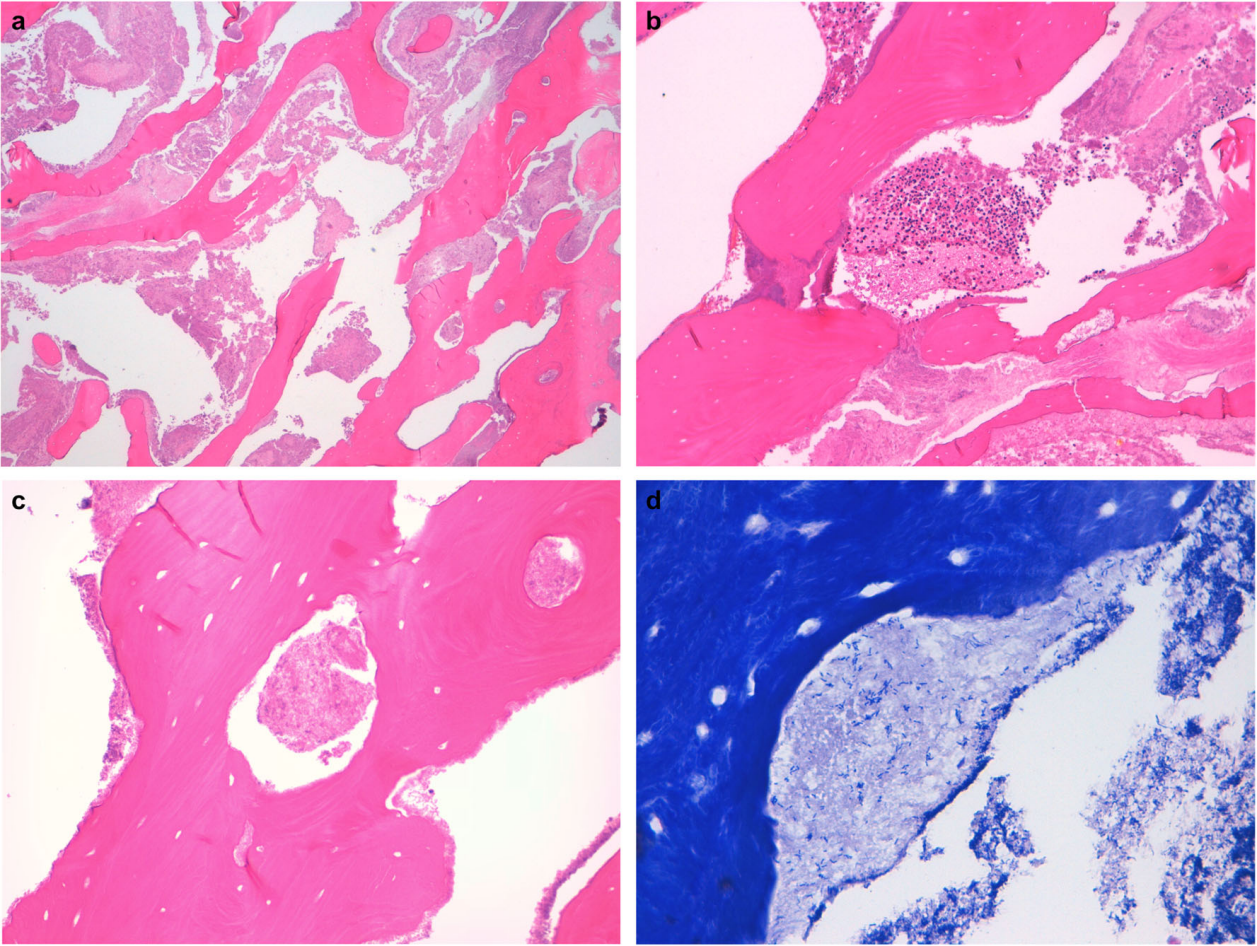
**a** Necrotic bone (H&E stain, 40× magnification). **b** Section with inflammatory focus, neutrophils (H&E stain, 100×magnification). **c** Section of necrotic bone, empty lacunae (H&E stain, 200× magnification). **d** Section of rod-shaped, gram-positive bacteria (Gram stain, 400× magnification)

Due to the aforementioned thrombopenia (platelet 50 × 10^3^/μl), the patient received platelet concentrate 2 h preoperatively in order to improve hemostasis during the surgical procedure and to prevent postoperative secondary bleeding. Afterwards, the sequestrotomy was performed under local anesthesia (1.7 ml articain hydrochloride 4% with epinephrine 1:200,000) followed by subgingival scaling using curettes of the adjacent root surface (Fig. 1c, d) and suturing in order to approach wound margins and to ease coagulation. Immediately after completion of the surgical treatment, both central maxillary incisors presented with significantly increased mobility. To prevent further impairment, temporary splint using a resin-based composite was placed (Fig. 1e). One day preoperatively, the patient received metronidazole (400 mg t.i.d.) which was continued upon completion of wound closure after 7 days. After uneventful healing, except slight residual gingival swelling, there were no signs of inflammation detectable (Fig. 1f). At final clinical visit, 5-week postoperative gingival swelling had entirely ceased and the gingival recession increased 2 mm at both incisors (Fig. 1g, h). Histopathological examination of the surgically removed tissue depicted in Fig. 1j confirmed the clinical findings by revealing necrotic bone with inflammatory cell infiltrate and gram-positive, rod-shaped bacteria (Fig. 2a–d).

## Discussion and conclusions

### AXL receptor tyrosine kinase—effects and inhibition

Bemcentinib (BGB324) is an inhibitor of the AXL receptor whose therapeutic effect on sAML is currently under investigation in clinical phase II trials [8, 9]. The AXL receptor tyrosine kinase is a member of the Tyro-3, AXL, and Mer (TAM) subfamily that can be almost ubiquitously found within many different tissues [5, 10–13]. Upon Gas6 activation, AXL mediates cell proliferation and migration through Ras/Raf/MEK/ERK and the Src signaling pathway as well as cell survival by S6, AKT, or JNK [10, 13, 14]. Regarding the immune system, AXL mediates efferocytosis, a reduced TLR-dependent inflammatory response, and natural killer cell activity [12, 14]. In the tumor immune microenvironment, AXL is often overexpressed by dendritic cells, NK cells, or macrophages, promoting epithelial to mesenchymal transition, tumor angiogenesis, resistance to chemotherapeutic and targeted agents, and decreased antitumor immune response [5, 13, 15, 16]. AXL activation in dendritic cells inhibits TLR and TLR-induced cytokine-receptor cascades [12] and reduces dendritic cell activity as well as complement T cell checkpoint cascade [16]. Activation of the tumor-associated macrophage receptor AXL by Gas6 or Protein S has shown to shift macrophage polarization, inducing pro-tumor m2 polarization and inhibiting pro-inflammatory m1 polarization [11]. Several studies confirm a TAM-related downregulation of m1 polarization leading to decreased secretion of pro-inflammatory cytokines such as interleukin-1 beta (IL-1β), interleukin-6 (IL-6), and tumor necrosis factor-α (TNF-α) [17–21]. Moreover, another study demonstrated increased expression levels of these cytokines in Gas6 or Protein S-inhibition models [20]. On the other hand, AXL signaling increases the polarization of m2-like macrophages. Under physiological conditions, the m2 phenotype is involved in maintenance of tissue homeostasis, consolidation of inflammatory processes, and wound healing through efferocytosis, angiogenesis, and secretion of anti-inflammatory cytokines [11, 14, 22, 23]. In the tumor microenvironment, however, m2-like functions are overexpressed via TAM regulation, ultimately leading to tumor angiogenesis [22, 24], impaired anti-tumor immunity, tumor growth, and metastasis [23, 25].

AXL inhibitors such as Bemcentinib counteract these numerous effects. By inhibiting the formerly altered macrophage polarization mentioned above, angiogenesis is impaired and the previously inhibited secretion of pro-inflammatory cytokines is increased consequently. These two effects might present a possible link to the complex pathogenesis of periodontal disease as occurred in this case. Actually, the aforementioned cytokines IL-1β and TNF-α are two key marker molecules in periodontitis that are known to play a crucial role in the development and maintenance of inflammatory tissue degradation and alveolar bone resorption [26, 27]. Interacting synergistically, they stimulate the expression of the receptor activator of nuclear factor-κB ligand (RANKL) on the surface of osteoblasts and stroma cells, thus promoting osteoclast formation and corresponding bone loss [28]. Additionally, the Bemcentinib-induced inhibition of angiogenesis in conjunction with the increased inflammatory component could favor a rapid necrotic course of periodontitis.

### Diagnosis—necrotizing periodontitis or medication-related osteonecrosis of the jaw

Necrotizing periodontal conditions as observed in the present case are of infectious etiology and strongly associated with impairment of the host immune system [29]. According to the current classification for periodontal and peri-implant diseases and conditions [29], necrotizing periodontitis (NP) is an acute inflammatory process of the periodontium characterized by necrosis/ulcer of the interdental papilla, gingival bleeding, pain, and rapid bone loss [30, 31]. As the clinical findings of this case were consistent with all these characteristics, the working diagnosis necrotizing periodontitis was adjusted accordingly.

However, considering the numerous aforementioned effects of Bemcentinib on signaling cascades and the associated anti-angiogenic and ultimately pro-inflammatory effects, the possible influence of the AXL inhibitor on the periodontal condition herein must be taken into account when determining a suspect diagnosis. Hence, the differential diagnosis of a medication-related osteonecrosis of the jaw was considered.

According to the 2014 AAOMS position paper, MRONJ is defined as “exposed bone or bone that can be probed through an intraoral or extraoral fistula(e) in the maxillofacial region that has persisted for more than eight weeks” in a patient that has currently or previously been treated with antiresorptive or antiangiogenic agents and shows no history of radiation therapy or obvious metastasis of the jaws [4].

Herein, there has been recorded no history of radiation therapy or obvious metastasis of the jaws and the AXL inhibitor Bemcentinib clearly has antiangiogenic effects but the exposure of jawbone did not persist for more than 8 weeks at the time of diagnosis. Currently, there exists considerably controversy on the AAOMS criteria and the definition as published by the International Task Force on ONJ in 2015 [32] specifically regarding the diagnosis of early stages and non-exposed cases of MRONJ that might precede clinical and radiographic evident sequestration and/or exposure of the jawbone [33]. Accordingly, one might argue that the necrosis of the alveolar bone as seen in the present case was already existent unperceived well before being clinically noticeable for the first time.

Moreover, histological analysis of the surgically removed bone clearly revealed colonization with gram-positive, rod-shaped bacteria. Regarding BRONJ, the colonization of the affected parts of the alveolar bone specifically with gram-positive, rod-shaped Actinomyces species is highly prevalent and was thus proposed to play a central etiologic role [34]. Apart from the microscopic analysis, bacteria have not been, however, further characterized in the present case.

## Considerations and conclusion

Regarding the pathogenesis of bone necrosis associated with MRONJ, different etiologic models have been intensively discussed considering mainly the effects of bisphosphonates [1, 4]. Currently, an over-suppression of bone turnover [4, 35–37] as well as the necrosis as final result of infection in combination with attenuation of the proliferation of various cell types which are centrally involved into the immune response are discussed [4, 38]. Moreover, MRONJ has been considered to result from ischemia triggered by medication-related antiangiogenic effects [4, 36], a general toxicity of the medication [4, 39–41], or medication-induced shifts in pH [42]. Oversuppression of bone turnover, comparable to osteoclast suppression by bisphosphonates or inhibition of RANK ligand mediated by Denosumab [1, 38, 43], is not yet known for the AXL inhibitor Bemcentinib. On the other hand, the above mentioned hypothesis of necrosis as the result of an underlying infection with an additional drug-related alteration of immune response from which a MRONJ may develop [34] seems just as conceivable as an impaired angiogenesis. In particular with respect to the previously mentioned shift of macrophage polarization by AXL and the assumed opposite shift by inhibition of AXL receptor, an excessive immune reaction induced by increased secretion of pro-inflammatory cytokines (m1-like phenotype induced) and simultaneously inhibited angiogenesis and efferocytosis (m2-like phenotype induced) is a possible mechanism of osteonecrosis of the jaw.

Furthermore, cellular toxicity caused by previously administered cytostatics might also play a role through modulation of the immune defense mechanisms, facilitating the necrosis. Nevertheless, it has to be emphasized that the necrosis developed within a comparably short period after the onset of the Bemcentinib regimen. Therefore, the AXL inhibitor might be at least the ultimate promoting factor. Finally, a reduction of the pH value might have been mediated through excessive inflammation and represents another possible mechanism of necrosis [42].

Taken together, it can be concluded that an irreversible necrosis and exposure of parts of the alveolar bone have developed under a short regimen of Bemcentinib in the present case consistent with the commonly accepted pathogenesis of MRONJ.

Oro-dental infections, especially periodontal disease, have been shown to significantly increase the risk for the development of MRONJ. Based on the present clinical case, we strongly recommend meticulous preventive dental measures also prior and during the administration of AXL inhibitors such as Bemcentinib.

## Data Availability

Not applicable.

## Abbreviations

μl: microliter
AAOMS: American Association of Oral and Maxillofacial Surgeons
AML: Acute myeloblastic leukemia
b.i.d.: Bis in die
BGB 324: Bemcentinib
BRONJ: Bisphosphonate-related osteonecrosis of the jaw
Fig.: Figure
h: Hour(s)
H&E: Haematoxylin and eosin stain
IL-1β: Interleukin-1 beta
IL-6: Interleukin-6
LMU: Ludwig-Maximilians-Universität
mg: Milligram
ml: Milliliter
mm: Millimeter
MRONJ: Medication-related osteonecrosis of the jaw
NK cells: Natural killer cells
NP: Necrotizing periodontitis
o.d.: Omni die
RANKL: Receptor activator of nuclear factor-κB ligand
sAML: Secondary acute myeloblastic leukemia
t.i.d: Ter in die
TAM: Tyro-3, Axl and Mer receptor tyrosine kinases
TLR: Toll-like receptor
TNF-α: Tumor necrosis factor-α
